# Adaptable Model Parameters in Non-Invasive Prenatal Testing Lead to More Stable Predictions

**DOI:** 10.3390/ijms20143414

**Published:** 2019-07-11

**Authors:** Juraj Gazdarica, Jaroslav Budis, Frantisek Duris, Jan Turna, Tomas Szemes

**Affiliations:** 1 Geneton Ltd., Bratislava 841 04, Slovakia; 2 Department of Molecular Biology, Faculty of Natural Sciences, Comenius University, Bratislava 841 04, Slovakia; 3 Slovak Centre of Scientific and Technical Information, Bratislava 811 04, Slovakia; 4 Comenius University Science Park, Comenius University, Bratislava 841 04, Slovakia

**Keywords:** non-invasive prenatal testing, statistical models, z-score

## Abstract

Recent advances in massively parallel shotgun sequencing opened up new options for affordable non-invasive prenatal testing (NIPT) for fetus aneuploidy from DNA material extracted from maternal plasma. Tests typically compare chromosomal distributions of a tested sample with a control set of healthy samples with unaffected fetuses. Deviations above certain threshold levels are concluded as positive findings. The main problem with this approach is that the variance of the control set is dependent on the number of sequenced fragments. The higher the amount, the more precise the estimation of actual chromosomal proportions is. Testing a sample with a highly different number of sequenced reads as used in training may thus lead to over- or under-estimation of their variance, and so lead to false predictions. We propose the calculation of a variance for each tested sample adaptively, based on the actual number of its sequenced fragments. We demonstrate how it leads to more stable predictions, mainly in real-world diagnostics with the highly divergent inter-sample coverage.

## 1. Introduction

Advanced prenatal screening is an important part of obstetric care. Current methods of prenatal testing, such as amniocentesis and chorionic villus sampling, involve invasive sampling of fetal material and are associated with a risk of miscarriage [1]. Non-invasive prenatal testing based on fetal DNA analysis from maternal circulation have been developed in order to prevent such risk. In 1997, the discovery of fetal cell-free DNA (cfDNA) in maternal plasma and serum has led to new developments in the field of non-invasive prenatal diagnostic, opening up new options in the field of obstetric research [2]. The fetal cfDNA is of placental origin [3], and it can be reliably detected from fifth week of gestation [4]. On average, fetal cfDNA comprises about 10% of all cfDNA fragments circulating in woman’s blood when sampling is done between 10 and 20 gestational weeks, but the dispersion is quite large [5]. The advance of massively parallel sequencing technologies combined with the rapid development of bioinformatic algorithms and tools brought about a new era of non-invasive prenatal identification of common fetal aneuploidies, now commonly known as non-invasive prenatal testing (NIPT) [6,7,8,9,10].

In this paper, we focus on the last part of the NIPT analysis, when a sample already underwent laboratory preparation, sequencing, and processing of the data (e.g., mapping, GC correction, etc.), namely the interpretation of the resulting data. Traditionally, a z-score—also termed normalized chromosomal value (NCV)—is used as a form of probabilistic measure of aneuploidies such as trisomies T13, T18, and T21. The form of this test is, there is a proportion of sequenced fragments from observed chromosome, and with them are the mean and standard deviation of the same value in a control population of euploid samples, respectively [11,12,13,14]; while the method proposed by [13] appears to be the best performing among the methods of this type. Model parameters and are typically trained on a euploid population. While this is sufficient for the samples that have similar depth of sequencing, we show that false positive (FP) and false negative (FN) calls may arise, if the tested sample differs in the sequencing depth from the training set. As it is now common to offer NIPT tests in various price ranges, the sequencing depth is what is usually scaled down in the cheaper tests. A possible but less practical solution to this would be to have multiple training sets for various sequencing depths.

In this paper, we propose a mathematical formula for calculation of model parameters, and, adaptively according to the actual sequencing depth of the diagnosed sample. Although the proposed model requires some parameters to be estimated or trained from the euploid or normal population, we show that these parameters are independent of the tested sample sequencing depth and can be estimated from training samples with relatively shallow sequencing depth.

## 2. Results

### 2.1. Low Coverage in Training Samples Leads to Underestimation of Z-Score in High Coverage Samples

**Figure 1:**
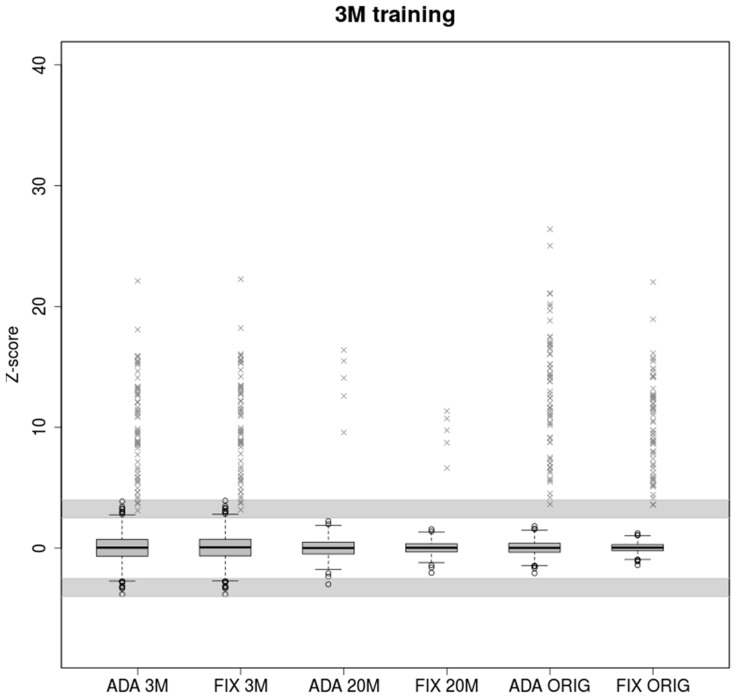
Boxplots of euploid NIPT samples for ADAVAR and FIXVAR models trained with samples subsampled to 3M reads uniquely mapped to autosomes. Models have been tested with equivalent samples subsampled to 3M (ADA 3M, FIX 3M) and 20M (ADA 20M, FIX 20M) respectively and with original (ORIG) read count of reads uniquely mapped to autosomes (ADA ORIG, FIX ORIG). Grey crosses represent trisomic samples. Samples in the grayed areas defined by ranges (2.5, 4) and (−4, −2.5) represent uninformative calls.

A lower number of reads leads to greater variability of observed chromosomal proportions between samples compared to deeply sequenced samples (Figure 1). The z-score is then lower in general, resulting in uninformative calls falling into the grey zone given by intervals (−4, −2.5) and (2.5, 4). Both models performed similarly when trained and tested on the samples with the same coverage (3M reads). 2840 euploid samples were tested, of which 39 and 42 fell into grey zone for ADAVAR (Adaptive Variance) and FIXVAR (Fixed Variance) models, respectively.

Parameters trained on low coverage samples naturally cannot fit deeply covered samples. The adaptively calculated standard deviation (SD) therefore performed markedly better in cases with a great difference between training and testing set. In the case of ADAVAR model, one euploid and none of the trisomic samples fell into a grey zone. At the hands of FIXVAR model, two trisomic and none of the euploid samples fell into a grey zone. We observed 1.44× higher z-score (*p* = 7.431 × 10^−7^) for ADAVAR model when trisomic sample were compared. Relatively higher variability of low coverage 3M samples thus leads to needless under-estimation of the z-scores in case of fixed model parameters.

We observed similar effect on real life samples with uneven coverage. Although all euploid samples were classified correctly in both methods, the z-scores of trisomic samples were significantly higher (1.24×, *p* = 0.0035) for the ADAVAR model.

### 2.2. High Coverage in Training Data Set Leads to Overestimation of Z-Score in Low Coverage Samples

**Figure 2:**
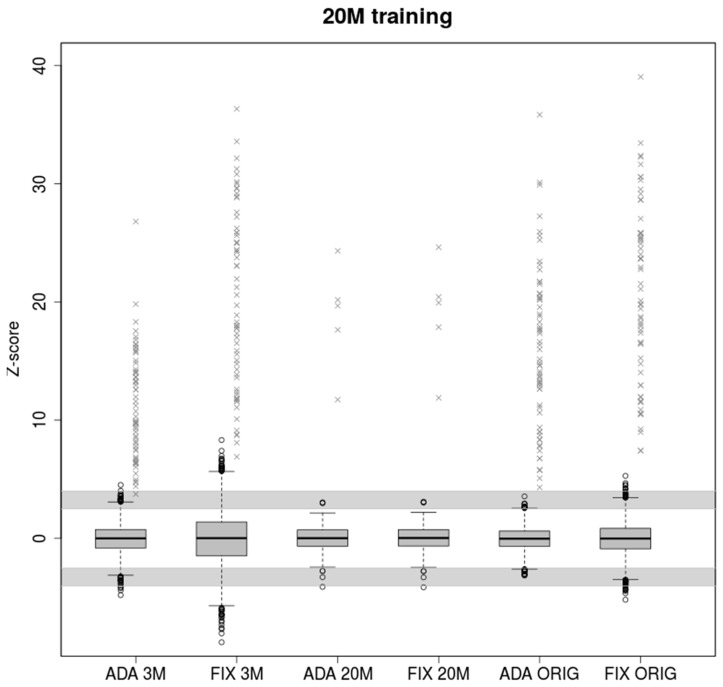
Boxplots of euploid NIPT samples for ADAVAR and FIXVAR models trained with samples subsampled to 20M reads uniquely mapped to autosomes. Models have been tested with equivalent samples subsampled to 3M (ADA 3M, FIX 3M) and 20M (ADA 20M, FIX 20M) respectively and with original read count of reads uniquely mapped to autosomes (ADA ORIG, FIX ORIG). Grey crosses represent trisomic samples. The grey areas defined by ranges (2.5, 4) and (−4, −2.5) represents uninformative calls.

Model parameters trained on 20M samples more accurately depict underlying chromosomal distributions than 3M samples due to the higher number of observed reads. Although the z-scores are higher for low covered samples, this led to more false positives exceeding the grey zone (Figure 2).

Parameters estimated using 20M training samples do not fit testing 3M samples properly. With a large number of testing data (5860 3M samples), we observed 1001 uninformative samples and 336 false positives (FP) in the case of FIXVAR model. This means almost every fourth test sample needs to be re-analyzed or evaluated as FP, which is not acceptable in clinical practice. Adaptive standard deviation reduced the number of uninformative results and FP calls from 1337 to 181 (6 FP), with a specificity 97%. On the other hand, in the case of ADAVAR model, the probability for false negative is slightly higher. Similarly, for 20M testing samples, the results are almost equal. All trisomic samples were classified correctly, and only seven euploid samples fell into a grey zone. 

When testing 5680 production samples with original read count, we observed a still large number of uninformative results in case of FIXVAR model, 303 (19 FP), but only 45 uninformative and none of the FP in case of ADAVAR model. In both cases, all trisomic samples were classified correctly.

### 2.3. Training on Samples with Uneven Coverage

**Figure 3:**
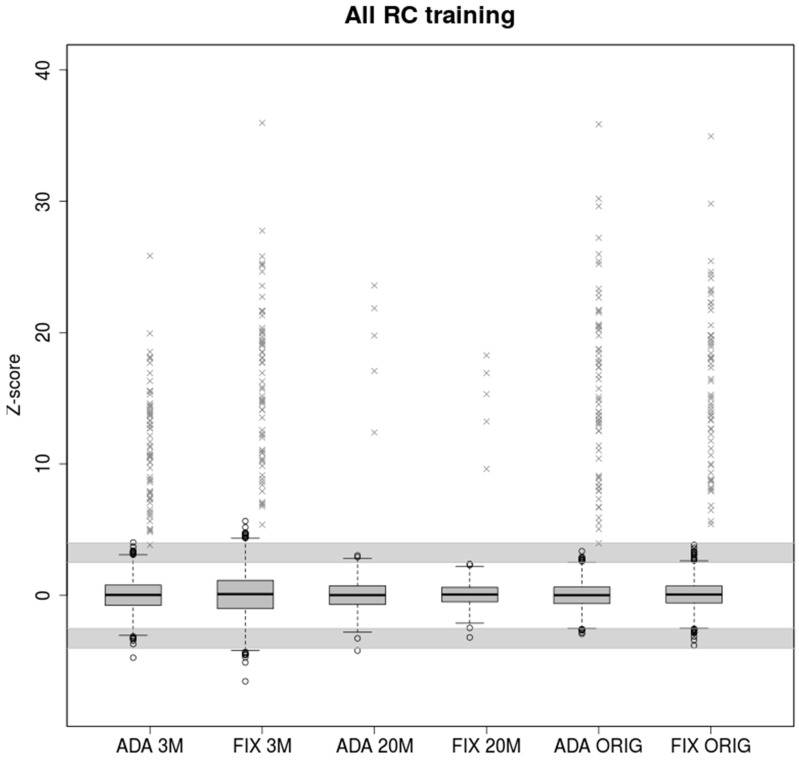
Boxplots of euploid NIPT samples for ADAVAR and FIXVAR models trained with samples contained original number of uniquely mapped reads. Models have been tested with equivalent samples subsampled to 3M (ADA 3M, FIX 3M) and 20M (ADA 20M, FIX 20M) respectively and with original read count of reads uniquely mapped to autosomes (ADA ORIG, FIX ORIG). Grey crosses represent trisomic samples. The grey areas defined by ranges (2.5, 4) and (−4, −2.5) represents uninformative calls.

Real parameters estimated from training samples with original read count provide enough information about the variability between the data. Also, in this case the two models have comparable results when testing real read count samples (Figure 3). Therefore, if enough training samples with wide read count range is available, both models provided high accuracy by testing. The adaptive standard deviation is valuable mostly in the limit case of the test samples.

In the case of testing 3M samples, FIXVAR model were found 330 uninformative calls (30 FP), which means too many samples for repeated analysis. On the other hand, the ADAVAR model is slightly more likely to report potentially false negative.

Z-scores values of healthy and trisomic samples displayed in Figure 1, Figure 2 and Figure 3 are available in supplementary_table_healthy_zscores.xlsx and supplementary_table_tris_zscores.xlsx (in Supplementary Materials), respectively.

## 3. Discussion

We propose an improvement for state-of-the-art methods used in NGS-based non-invasive testing based on adaptive model parameters that are calculated for each sample separately. The method is based on theoretical properties of underlying distributions that provide estimates of variance in random draw from multinomial distribution. We have shown that those estimates differ from observed variance by constant factor, that may be easily incorporated into the calculation and improves the model beyond the level of current best methods used in clinical practice.

We tested the limitations of the commonly used method FIXVAR and the proposed ADAVAR method on boundary sequencing depths. We have also tested these methods on real data sets with uneven sequencing depths. Although the new method did not greatly exceed the current methods in ordinary cases, its benefits are in borderline cases.

As we have shown in the results, when training on low read count followed by testing on many times higher number of reads, ADAVAR provided significantly higher z-score values than FIXVAR (Figure 1). Higher coverage is typically required for more thorough predictions, for example, in the case of repeated analysis, detection of mosaicism, or partial chromosomal aberrations.

As a result, the number of false negative calls is greatly reduced without increasing the number of false positive calls. FIXVAR method also performs poorly, when the model parameters are trained on samples with higher read count values than testing samples. Underestimation of variance in tested samples leads to a high amount of false positive calls. We have shown that the new method is able to partially correct these ineligible clinical results with respect to the number of reads and thus avoid the high number of false positives. This is the case when a sequenced sample has lack of reads which can be caused by several factors, for example, a large number of sequenced samples, insufficient concentration of DNA fragments, or uneven distribution of pooled samples to be sequenced.

In the article, we pointed out the shortcomings of current methods and their partial correction by our method. Although the new method ADAVAR has not overcome standard methods in all cases, it still has benefits in testing of samples with highly divergent coverages, where this method leads to a lower number of false positive and false negative calls.

## 4. Materials and Methods 

### 4.1. Sample Acquisition

Altogether, we have collected 6117 samples with singleton pregnancy, of which 6053 were negative, while 64 were confirmed for trisomy of chromosome 21 (T21). In each case were positive results confirmed by amniocentesis. Negative samples were, however, not confirmed by any additional gold standard method. Data analyses reported here, were, on the other hand performed only on samples originally analyzed with a sufficient time interval to know, from a clinician feedback following the delivery, whether any false negative results occurred. Note that the sample set does not contain samples that we were not able to resolve (such samples were either repeated or declined to report). The samples were predominantly of Slovak and Czech origin. All women participating in this study gave informed written consent consistent with the Helsinki declaration. Ethic approval: Etická komisia Bratislavského samosprávneho kraja (Ethical commission of self-governing region of Bratislava), approval number: 07507/2018/HF, approval date: 11 June 2018.

### 4.2. Sample Preparation and Sequencing

Blood from pregnant women was collected into EDTA tubes and kept at 4 °C temperature until plasma separation. Blood plasma was separated within 36 h after collection and stored at −20 °C unit DNA isolation. DNA was isolated using Qiagen DNA Blood Mini kit (Hilden, Germany). Standard fragment libraries for massively parallel sequencing were prepared from isolated DNA using an Illumina TruSeq Nano kit (San Diego, CA, USA) and a modified protocol described previously [15]. Briefly, to decrease laboratory costs, we used reduced volumes of reagents what were compensated by 9 cycles of PCR instead of 8 as per protocol. Physical size selection of cfDNA fragments was performed using specific volumes of magnetic beads in order to enrich fetal fraction. Illumina NextSeq 500/550 High Output Kit v2 (San Diego, CA, USA) (75 cycles) was used for massively parallel sequencing of prepared libraries using pair-end sequencing with read length of 2×35bp on an Illummina NextSeq 500 platform (Available online: https://www.illumina.com/).

### 4.3. Mapping and Read Count Correction

The first part of analysis was performed as described previously in [15,16,17]. Sequencing reads were aligned to the human reference genome (hg19) using Bowtie 2 algorithm [18]. The first stage of data processing was carried out as in [15,18]. NextSeq-produced fastq files (two per sample) were directly mapped using the Bowtie 2 algorithm with --very-sensitive option. Reads with mapping quality of 40 or higher were retained for further data processing. For some of our analyses, a uniform random selection of only some amount of mapped reads (alignments) was chosen for further processing. Next, for each sample, the unique reads were processed to eliminate the GC bias according to [19] with the exclusion of intra-run normalization. Briefly, for each sample the number of unique reads from each 20 kbp bin on each chromosome was counted. With empty bins filtered out, the locally weighted scatterplot smoothing (LOESS) regression was used to predict the expected read count for each bin based on its GC content. The LOESS-corrected read count for a particular bin was then calculated as $RC_{cor}=RC-\left|RC_{loess}-RC_{avg}\right|$, where $RC_{avg}$ is the global average of read counts through all bins, $RC_{loess}$ is the fitted read count of that bin, and RC is its observed read count. 

To remove genomic regions with common structural differences, the LOESS-corrected bin counts were transformed into a principal space. The first component represents the highest variability across individuals in the control set. To normalize the sample, bin counts corresponding to a predefined number of top components were removed to reduce common noise in euploid samples [20,21]. Vector of corrected number of reads per autosomes was used for z-score calculations.

### 4.4. FIXVAR (Fixed Variance) Z-Score Calculation

The reference z-scores of samples were calculated as normalized chromosome values (NCV) according to [13]. Given our training set, the optimal reference chromosomes with respect to the coefficient of variation were determined to be 1, 4, 8, 10, 19, and 20 for trisomy 21 [13]. Similarly to [7], samples scoring 4 and higher were considered trisomic, while samples scoring 2.5 or lower were considered euploid. The range (2.5, 4) was considered uninformative. We will refer to these NCV values as reference z-scores or $Z_{FIX}$ and to this type of z-score calculation (ratio of chromosomes) as FIXVAR model.

### 4.5. ADAVAR (Adaptive Variance) Z-Score Calculation

#### 4.5.1. Motivation

Consider a multinomial distribution given by (n,p1,p2,…,p22) as a model for mapping of n sequenced reads to autosomes (we omitted sex chromosomes due the different mapping ratio for male and female fetuses). The numbers $p_{i}$ are associated with proportion of reads mapped to the *i*th autosome, and are largely determined by structure and composition of the chromosome, such as its length, GC content, repeat sequence distribution and so on. However, it was observed that there exist differences between healthy individuals on sub-autosomal level (typically copy number variations or CNVs) large enough to skew the theoretical random draw from multinomial distribution (Kucharik 2019, under review). Even though we omitted those parts of the genome that exhibited such variations frequently, individual deviations from the central model, presumably due to random individual CNVs, still exceeded the statistical errors expected from the assumed multinomial distribution. Still, an approximation of the numbers $p_{i}$ can be obtained through a sufficiently large and diverse sample of population even though a population-universal multinomial mapping model is unlikely to exist. We showed that with sufficient corrections, the approximate model can still be useful, and it outperforms FIXVAR model in certain cases.

#### 4.5.2. Definition

Formally, the model is defined as follows. Let a set of random variables $X=\left(X_{1},X_{2},\ldots ,X_{22}\right)$ have joint multinomial distribution given by (n,p1,p2,…,p22). The instance of this random variable represents counts of reads mapped to autosomes for a given biological sample. Let $u,v\in {\left\{0,1\right\}}^{22}$ be two binary vectors such that $\sum u_{i}>0$, $\sum v_{i}>0$, and $u_{i}v_{i}=0$ for all i. The vector u selects an aneuploid autosome (thus, we have $\sum u_{i}=1$), and the vector v selects reference autosomes. Because we do not want the aneuploid or potentially aneuploid autosome to be in the reference set, some other restrictions further apply, namely $u_{i}v_{i}=0$ for all i (i.e., the trisomic autosome is not in the reference set) and $v_{13}=v_{18}=v_{21}=0$ (neither are three common trisomic autosomes). The reference autosomes can be found by many methods, for example, through minimization of coefficient of variation as in [13].

Let Y be a new scalar random variable defined as

(1) $$Y=\frac{X\cdot u}{X\cdot v}$$

where · stands for the scalar product. Observe that this is the model of chromosome ratio from [13]. With p=(p1,p2,…,p22), $q_{1}=p\cdot u$, and $q_{2}=p\cdot v$, ref. [22] showed that for sufficiently large n the following approximations of mean and standard deviation of Y holds

(2) $$\mu_{ADA}\left(Y\right)\approx \frac{q_{1}}{q_{2}}+\frac{q_{1}}{q_{2}^{2}n}$$

(3) $$\sigma_{ADA}\left(Y\right)\approx \sqrt{\frac{1}{n}{\left(\frac{q_{1}}{q_{2}}\right)}^{2}\left(\frac{1}{q_{1}}+\frac{1}{q_{2}}\right)}$$

where n is the total autosomal read count of a given sample (more robust approximations can be found in the paper). Observe that while the numbers $q_{1}$ and $q_{2}$ are determined by the reference set, the number n changes with each test sample. Thus, the mean and standard deviation is automatically adjusted for variable sequencing depth. Finally, we can calculate the sample’s z-score, an analogue to $Z_{FIX}$, as

(4) $$Z_{ADA}=\frac{Y_{sample}-\mu_{ADA}\left(Y\right)}{\sigma_{ADA}\left(Y\right)}$$

#### 4.5.3. Additional Bias Correction

As we pointed out before, this central ADAVAR model does not represent a general euploid pregnancy in sufficient detail, presumably because of the random individual CNVs. Hence, the model needs to be modified before it can be used for z-score calculation.

**Figure 4:**
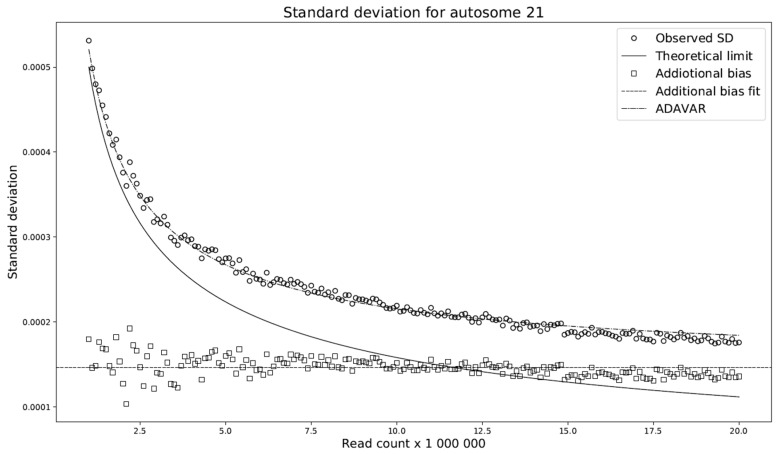
Comparison of standard deviations with respect to the selected read count among the individual models. Observed SD mean observed standard deviation for FIXVAR model. The theoretical limit denoting calculated standard deviation for ADAVAR model and constant dash line represents additional bias across various read counts used by ADAVAR. The prediction of the standard deviation of the theoretical limit (solid line) is not in good agreement with the reference/observations (circles), presumably due to individual CNVs. Observe that the difference between reference/observations (circles) and predicted standard deviations (theoretical limit) is approximately constant throughout the whole range (squares), and adding the mean of the differences to the predicted standard deviation yields a very good approximation of the observations (dash-dot line).

This modification compares the standard deviations with respect to the selected read count among the individual models. We have shown (Figure 4) that the difference between these deviations is almost constant across any read count setting. Let this constant be denoted by c. We set c to be the average of the deviation differences. The theoretical model ADAVAR then has a standard deviation defined as

(5) $$\sigma_{ADAc}=\sqrt{\sigma_{ADA}^{2}+c^{2}}$$

**Figure 5:**
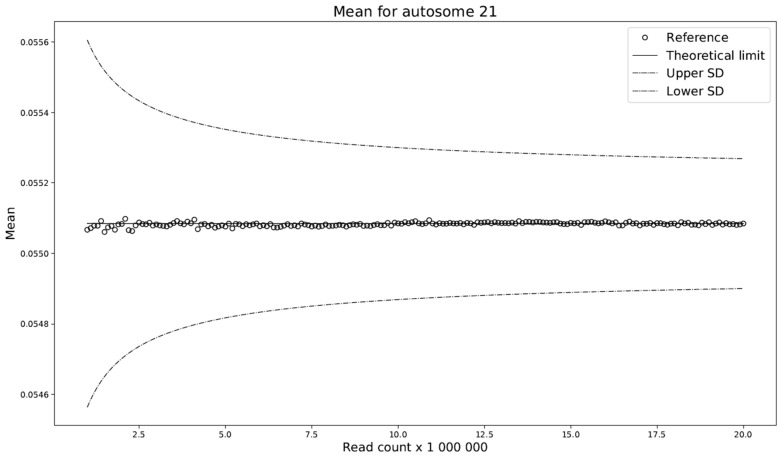
Prediction of the mean of the theoretical limit (solid line) is in good agreement with the observations (circles). The dashed lines represent one corrected standard deviation above and below the predicted mean.

Furthermore, prediction of the mean of the ADAVAR model and the observations agree (Figure 5) and no further correction is needed.

Then the sample’s z-score is given by

(6) $$Z_{ADAc}=\frac{Y_{sample}-\mu_{ADA}\left(Y\right)}{\sigma_{ADAc}\left(Y\right)}$$

## Supplementary Materials

Supplementary_methods.docx [23]—Details of training and testing, supplementary_table_healthy_zscores.xlsx – z-scores values of healthy samples showed in Figure 1, Figure 2 and Figure 3, supplementary_table_tris_zscores.xlsx - z-scores values of trisomic samples showed in Figure 1, Figure 2 and Figure 3. The following are available online at https://www.mdpi.com/1422-0067/20/14/3414/s1.

## Abbreviations

NIPT	Non-invasive prenatal testing
FP	False positive
FN	False negative
T21	Trisomy of chromosome 21
NCV	Normalized chromosome values
FIXVAR	Fixed variance model
ADAVAR	Adaptive variance model
ADA	Abbreviation of ADAVAR
