# Supplementary material for: Adaptable Model Parameters in Non-Invasive Prenatal Testing Lead to More Stable Predictions
Source: Int J Mol Sci. 2019 Jul 11;20(14):3414. doi: 10.3390/ijms20143414 (PMC6678500; doi:10.3390/ijms20143414)
Supplement: Supplementary file 1 [file ijms-20-03414-s001.zip › Supplementary_methods.docx]

Supplementary material

Training & testing

A training set of pregnant women with single euploid foetus was used for the estimation of the ADAVAR and FIXVAR model. In this study we firstly trained model parameters for limiting number of reads uniquely mapped to autosomes, specifically samples for training were subsampled to 3,000,000 reads (3M dataset). We also subsampled samples with sufficient coverage to 20,000,000 reads (20M dataset). Finally, we trained samples with uneven number of uniquely mapped reads without subsampling (original samples).


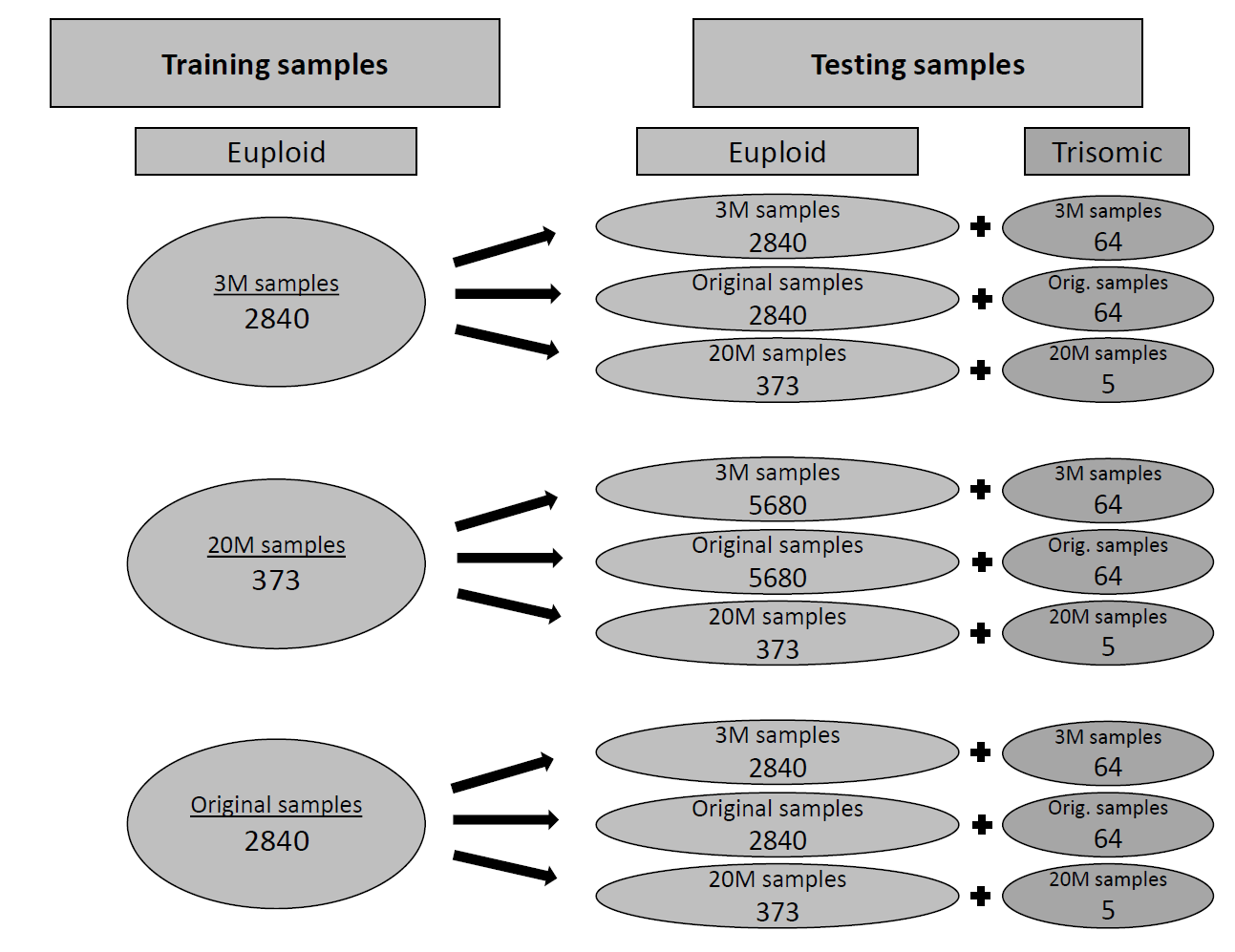


**Supp. figure 1**: Graphic scheme for training and testing sample allocation

For 20M case, from 746 samples with at least 20M reads uniquely mapped to autosomes one half was selected for training (373 samples) and one half for testing (373 samples). All training samples were first subsampled to 20M to reduce the between-sample variability induced by variations in sequencing depth. For each training sample, we calculated the fractional genomic representation [23] of the autosomal counts from which the mean fractional representation of each autosome was calculated, resulting in the numbers which were used as the central ADAVAR model. 5680 samples with original RC > 3M (included samples with RC >20M) and the same 5680 samples but subsampled to 3M were used for testing. In both testing datasets, samples with RC > 20M used in training process were excluded.

We repeated the same process for samples with at least 3M reads per sample, where samples with RC > 20M used for 20M testing were excluded from all data, resulting in 5680 samples, from which 2840 samples were subsampled to 3M used for training, resulting in numbers, the rest of them were used for testing. Similar to training with 20M samples, 373 samples with original RC > 20M and the same 373 samples but subsampled to 20M were used for testing.

Finally, from 5680 samples with at least 3M reads uniquely mapped to autosomes were 2840 samples selected for training where numbers were determined. Complementary 2840 samples with original RC > 3M and the same 2840 samples but subsampled to 3M were used for testing. 373 samples with original RC > 20M subsampled to 20M were used for testing.

For aneuploid testing we used 64 samples with trisomy 21 of which 5 samples with coverage more than 20,000,000 reads.
